# Bibliometric analysis of breast cancer-related lymphedema research trends over the last 2 decades

**DOI:** 10.3389/fonc.2024.1360899

**Published:** 2024-02-20

**Authors:** Jinghui Huang, Jiamin Li, Ying Li, Lele Huang, Bai Li, Feng Huang, Can Lv, Fanfu Fang

**Affiliations:** ^1^ Department of Rehabilitation Medicine, The First Affiliated Hospital of the Naval Medical University, Shanghai, China; ^2^ School of Health Science and Engineering, University of Shanghai for Science and Technology, Shanghai, China

**Keywords:** breast cancer-related lymphedema, CiteSpace, VOSviewer, bibliometric analysis, research trends

## Abstract

**Objective:**

As breast cancer cases rise globally, post-mastectomy lymphedema garners increasing scholarly attention. This study aims to conduct a comprehensive bibliometric analysis of Breast Cancer-Related Lymphedema (BCRL) research from 2003 to 2022, identifying trends and providing global research insights for future studies.

**Method:**

The literature for this analysis was extracted from the Web of Science (WoS) Core Collection, encompassing 1199 publications, including 702 articles and 101 reviews, totaling 803. Using advanced bibliometric tools such as VOSviewer and CiteSpace, quantitative and visual analyses were performed to map collaboration networks, research clusters, and emerging trends. The search strategy included specific terms related to lymphedema, breast cancer, and BCRL, ensuring a comprehensive representation of the research landscape.

**Results:**

The bibliometric analysis revealed a steady increase in BCRL publications over the studied period, reaching a peak in 2018. The United States emerged as the leading contributor to BCRL literature, with China also demonstrating a significant presence. Collaboration networks were visualized, showcasing the interconnectedness of institutions and researchers globally. Key research hotspots identified include preventive strategies, complex decongestive therapy, and reconstructive interventions.

**Conclusion:**

In conclusion, this pioneering bibliometric analysis provides a comprehensive overview of BCRL research trends and collaborations globally. The findings contribute valuable insights into the evolution of the field, highlighting areas of focus and emerging research themes. This study serves as a foundational resource for researchers, clinicians, and policymakers, fostering evidence-based practices and interventions for BCRL in the future.

## Introduction

Breast cancer stands as one of the most prevalent malignant neoplasms affecting women today. According to the latest data from the Centers for Disease Control and Prevention (CDC), breast cancer constitutes 31% of new cases among female tumor patients in the United States, claiming the highest incidence rate (1, 2). China is witnessing a substantial increase in the rate of breast cancer incidence, with a consistent rise observed in recent years (3). The advent of advanced medical technologies has led to a noteworthy improvement in the survival rates of breast cancer patients. Correspondingly, the mortality rate associated with breast cancer has experienced a consistent decline, culminating in a projected five-year survival rate of 90% for breast cancer survivors in the United States by 2022 (1). This progress underscores the positive impact of ongoing advancements in medical science on the prognosis and outcomes of individuals grappling with breast cancer.

Breast cancer treatment, encompassing axillary lymph node biopsy, clearance, and radiation therapy, can induce disruptions in lymphatic circulation, leading to secondary lymphedema in the upper extremities. Postoperative upper limb lymphedema, commonly referred to as BCRL, significantly impacts the long-term quality of life for survivors and stands as one of the most prevalent postoperative complications following breast cancer interventions. BCRL exerts a profound influence on the well-being of breast cancer patients (4). Manifestations of this condition include limb swelling, pain, and functional limitations, contributing to a substantial decline in the overall quality of life. The persistent symptoms associated with BCRL further give rise to adverse psychological outcomes, including heightened levels of anxiety and depression among affected individuals (5, 6).

Owing to the existing lack of uniform and standardized diagnostic and measurement criteria for BCRL, coupled with the continual introduction of novel assessment instruments and devices, the clinical conditions of patients participating in various studies, encompassing surgery, radiotherapy, and extended radiotherapy, have resulted in a broad spectrum of reported incidence rates. Secondary lymphedema following breast cancer treatment has been documented in reports spanning a wide range, from 2% to 83%. Several investigations have highlighted that the risk of lymphedema is markedly lower in cases of breast-conserving surgery compared to radical mastectomy, sentinel lymph node biopsy in contrast to axillary lymph node dissection, and regional lymph node irradiation versus extended irradiation (7, 8). The incidence of BCRL varies from 3% to 36.7% with regional lymph node irradiation and 10% to 50% following axillary dissection (9, 10). Patients with BCRL commonly report a range of symptoms, with 88% experiencing swelling, 72% tightness, 60% heaviness, and 40% numbness (11). The presence of BCRL significantly diminishes the quality of life for affected individuals, impacting body image and contributing to psychological issues such as depression and anxiety. Studies examining the economic burden of BCRL have underscored the occurrence of recurrent infections, substantially increasing healthcare expenditures for patients and complicating the treatment process (12, 13).

In recent years, there has been a gradual increase in the number of publications addressing BCRL, presenting a challenge for scholars seeking to swiftly comprehend the key issues and prevailing trends in BCRL research. Bibliometric analysis is recognized as a quantitative statistical tool that delineates the knowledge structure and identifies keyword trends within a specific research domain (14). Employing bibliometrics allows researchers to generate a comprehensive overview of the global distribution of countries, institutions, authors, and journals engaged in a particular research topic. This approach enables the visualization of hot topics and emerging trends in BCRL research, providing clinicians with evidence-based decision-making capabilities. Recognizing the need for a more robust quantitative analysis of BCRL-related literature, this study aims to delineate the global scientific output of BCRL research from 2003 to 2022. The study further seeks to provide quantitative insights into countries, institutions, journals, authors, and keywords, with the overarching goal of summarizing research hotspots and trends in BCRL. Anticipating that this analysis will offer valuable research references for future scholars, our objective is to contribute to the advancement of knowledge in this critical area of medical research.

## Methods

### Research strategy and data collection

The literature for this paper was sourced from the Web of Science (WoS), a repository encompassing over 20,000 high-quality and influential scholarly journals spanning 250 disciplines globally. The database, equipped with comprehensive citation indexing records, offers a robust platform for data mining and co-citation analysis (15). Recognized for its authority as a data source in bibliometric analysis, the Web of Science stands as a preferred choice due to its extensive coverage of scholarly publications, making it the primary selection for bibliometric investigations (16, 17).

**Figure 1:**
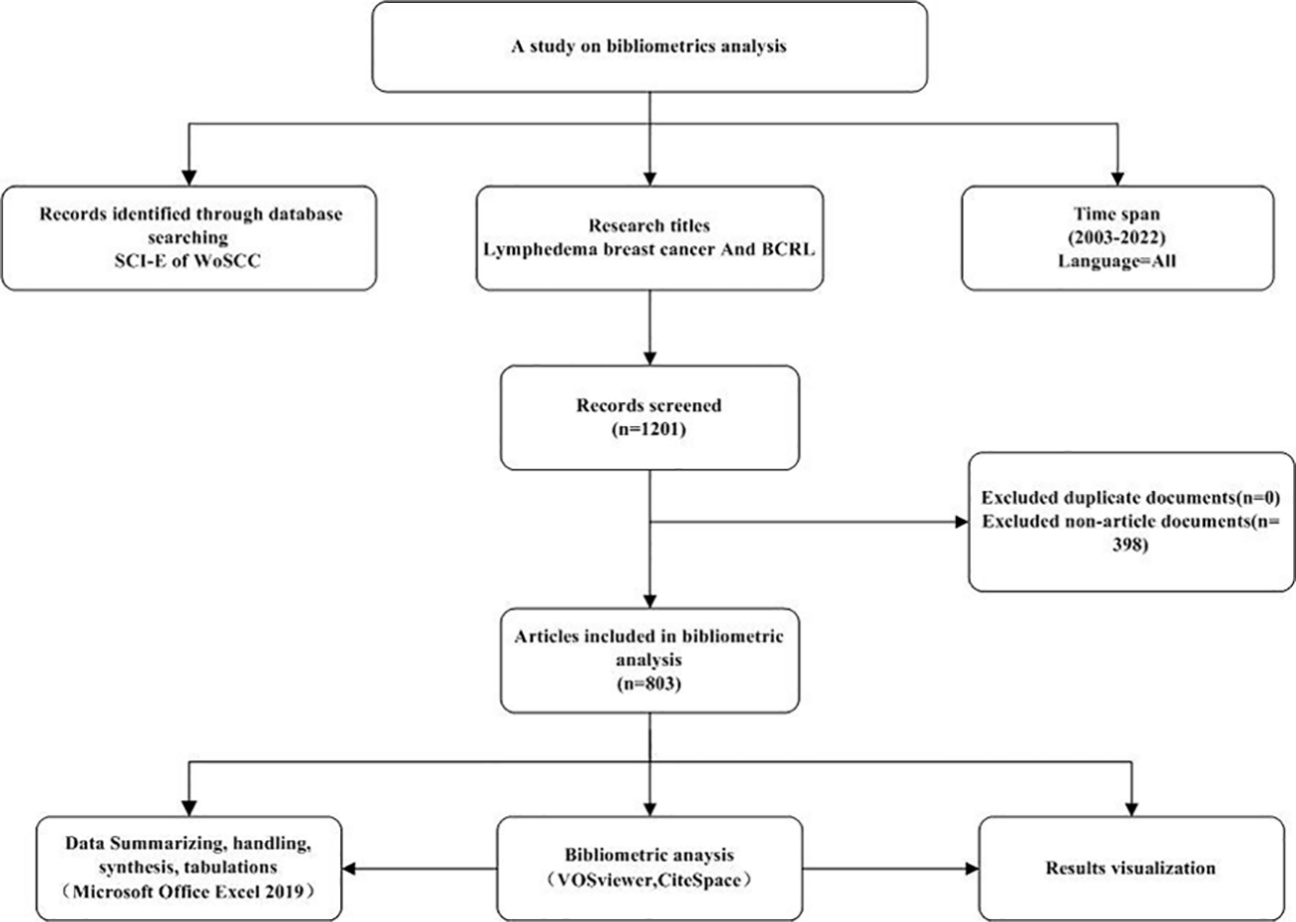
Flow diagram of searching process.

The main focus of our study is the research trends in BCRL over the past 20 years. Therefore, we conducted a systematic search and retrieval of relevant publications from January 2003 to December 2022 using the Science Citation Index Expanded (SCI-E). The search strategy employed the following parameters: TI= (lymphedema breast cancer OR BCRL), language=all, document type=articles or reviews. Additionally, we screened titles and abstracts for inclusion and exclusion criteria. Inclusion criteria were as follows: (1) Published between January 1, 2003, and December 31, 2022; (2) Limited to articles and reviews; (3) Content primarily focused on BCRL. Exclusion criteria were as follows: (1) Duplicate publications; (2) Non-article documents (such as book reviews, notices, editorials, conference abstracts, conference papers, letters, etc.). Two authors independently classified the extracted data. In cases of disagreement, a third author participated in discussions to reach a consensus. As of December 31, 2022, a total of 803 publications were successfully retrieved from the specified databases. The literature search methods and screening process are illustrated in the flow diagram of the searching process (**Figure 1**).

This study included a total of 803 papers, and the records were exported as “plain text files.” The export format comprised “complete records and cited references,” and the files were saved in “download_txt” format.

### Knowledge visualization analysis

This study employed CiteSpace, VOSviewer, and Microsoft Excel 2021 for quantitative and visual analysis. VOSviewer, a tool for analyzing key information from a substantial number of publications, was utilized to construct collaborative, co-citation, and co-occurrence networks (18). Node size in these networks indicates the number of publications, line width represents the strength of relationships, and node color signifies distinct clusters or cycles. CiteSpace, focusing on the fundamental analysis of scientific literature, serves as a visual analytics tool expanding into the realms of data visualization and scientometrics. In this study, CiteSpace was employed to generate knowledge networks, citation paths, and to detect bursts in references and keywords (19). Evaluation metrics included citation bursts, co-citation citation clustering networks, and keyword bursts. The emergence of keyword bursts or citations implies frequent appearance or citation over time, signifying topics that have garnered significant attention from researchers. As such, these bursts can be considered research hotspots or frontiers (20). Leveraging these indicators, crucial topics, recent advancements, and emerging trends in the field were effectively identified.

In this study, VOSviewer (1.6.19) and CiteSpace (6.2.2) software were used to visualize and analyze the BCRL-related literature in the WOSCC database and to draw a knowledge map, aiming to understand the current status of research, research hotspots, and development trends in the field, and to provide a reference for future research.

## Results

### Publication activity

**Figure 2:**
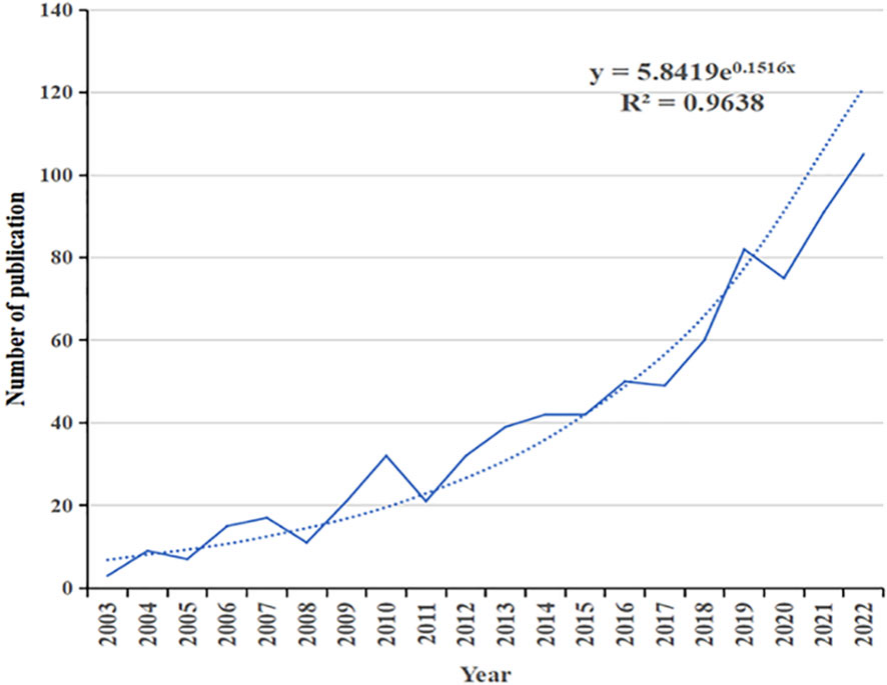
Annual publication outputs and growth prediction from 2003 to 2022.

A total of 1,199 publications spanning the period from 2003 to 2022 were identified, comprising 702 articles and 101 reviews, totaling 803. The articles and reviews cited a cumulative literature count of 7,067, excluding self-citations, which amounted to 6,297. The total number of citations reached 24,130, with co-citations removed, resulting in a net of 17,611 citations, averaging 30.05 citations per publication. The trend in annual publications and citations demonstrates a consistent increase, as depicted in **Figure 2**. The average number of citations per paper in WoSCC rose from 0.33 in 2003 to 29.31 in 2022. The majority of these publications are articles, with 105 articles and a cumulative citation count of 3,078 in 2022. The annual count of publications and citations serves as an indicator of research trends and field impact, respectively. Notably, 413 articles, equivalent to 51.43% of the total, were published in the past 5 years, highlighting the global research attention garnered by BCRL as a prevalent complication of postoperative breast cancer.

The surge in the number of articles published in this research domain aligns approximately exponentially with the predictive model equation y = 5.8419e^0.15165x^, where Y denotes the number of publications, and X denotes the year. The R^2^ value of 0.9638 indicates a robust fit to the curve, signifying a sustained research interest and a distinct research foundation in this area.

### Analysis of countries/regions

**Figure 3:**
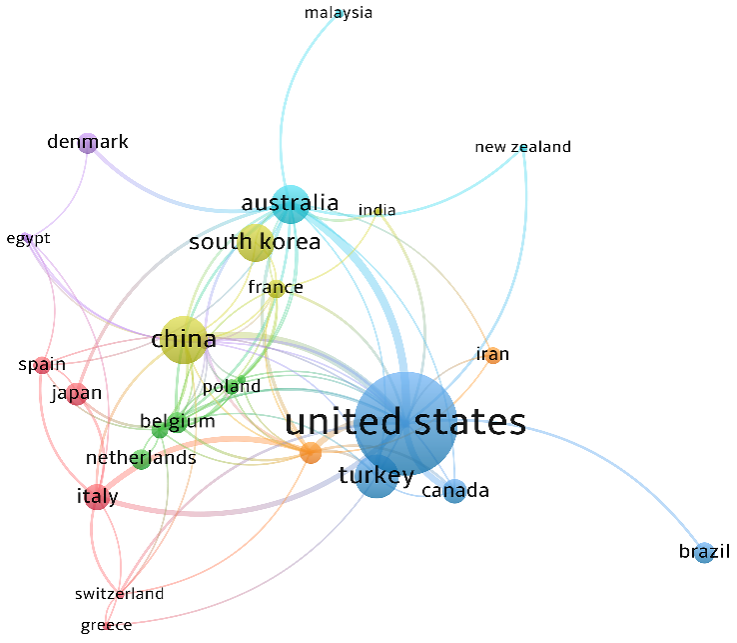
Collaboration network of countries or regions.

In **Table 1**, the analysis of country-specific publication volumes reveals that the United States leads in the number of BCRL-related papers published over the past two decades, with China emerging as the developing country with the highest publication count. The United States also ranks first in terms of citations, accumulating 12,642 citations, while Australia takes the second position with 1,913 citations. Canada claims the top spot in average citations, boasting an impressive 52.069, suggesting that Canada is emerging as a noteworthy contributor. China secures the second-highest publication count, contributing 1,421 cited articles with an average citation of 16.5233. Italy stands out for having the earliest average publication time, indicating its early involvement in relevant research. A holistic analysis considering publications, links, citations, and average citations collectively underscores the United States' prominent position in this research domain. **Figure 3** shows the collaborative network between countries.

**Table 1:** Top 10 countries based on the total number of publications for 2003 to 2022.

Country	Publications	Citations	Avg. citations	Avg. Pub. Year
United States	286	12642	44.2028	2018.6163
China	86	1421	16.5233	2018.5405
Turkey	74	1043	14.0946	2016.5902
Australia	61	1913	31.3607	2017.3966
South Korea	58	891	15.3621	2018.3438
Italy	32	905	28.2812	2012.2759
Canada	29	1510	52.069	2018.4231
Japan	26	365	14.0385	2014.68
United Kingdom	25	918	36.72	2017.0833
Belgium	24	376	15.6667	2018.6163

Avg. pub. Year, Average publication Year; Avg. citations, Average citations.

### Analysis of institutions

**Figure 4:**
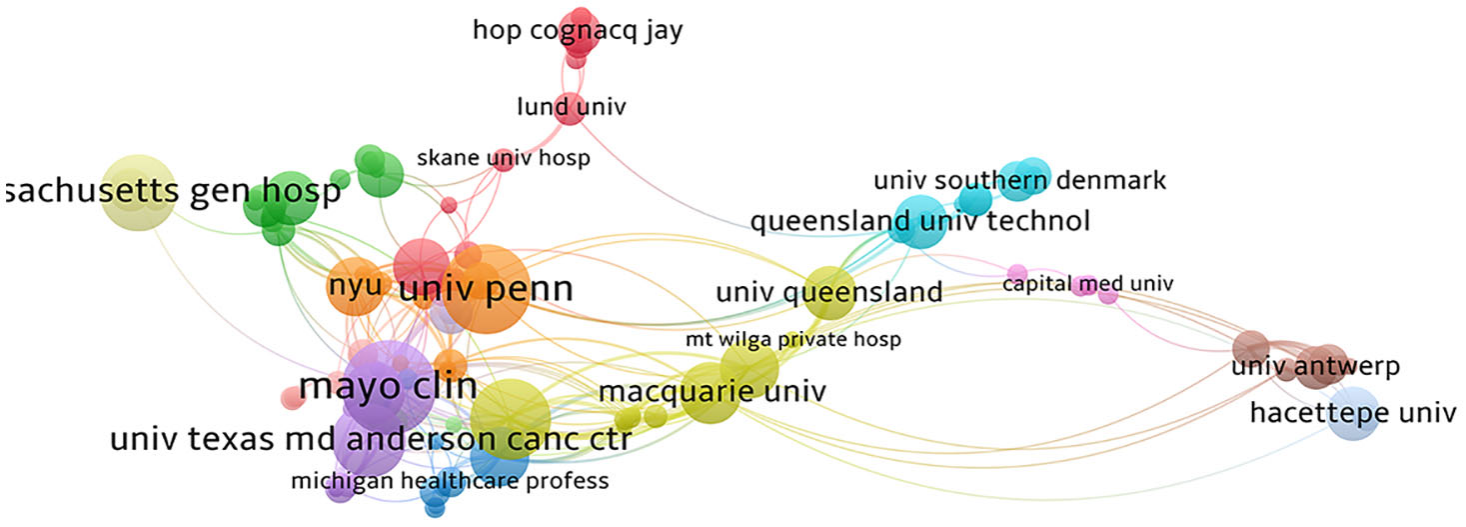
Collaborative network of institutions.

In **Figure 4**, the visualization depicts the volume of publications and various clusters of issuing organizations. Node size corresponds to the number of publications, line width indicates relationship strength, and node color signifies distinct clusters or cycles. The institutional analysis graph reveals that 110 institutions have contributed more than 3 publications each, collectively contributing 779 articles, which constitutes 97.01% of the total document count. The leading 10 institutions in terms of article numbers have published a combined total of 222 articles, representing 27.65% of the overall count. Most of the top 10 institutions, primarily research and clinical institutions, are based in the United States, maintaining robust collaborative relationships. The institution with the highest number of articles is Mayo Clinic, contributing 35 articles, followed by the University of Missouri and the University of Pennsylvania with 30 articles each. While China ranks second globally in terms of the overall number of articles, the issuing institutions appear relatively dispersed, with smaller article counts per institution, suggesting a need for strengthened collaboration.

### Analysis of journals

**Figure 5:**
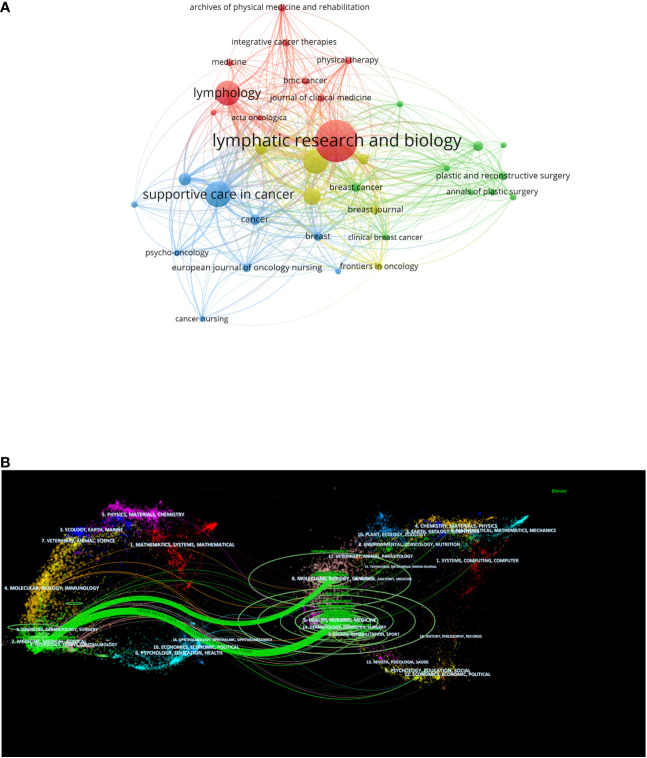
**(A)** Bibliographic coupling analysis of high publication volume journals, visualization maps. **(B)** The dual-map overlay of journals.

Academic journals serve as the conduit through which researchers disseminate their findings, playing a crucial role in reflecting the quality of research. To ensure a comprehensive analysis for mapping, journals with six or more articles were selected, resulting in the inclusion of 35 eligible journals. Lymphatic Research and Biology emerged as the journal with the highest article count, boasting 94 articles, equivalent to 11.71% of the total, followed by Supportive Care in Cancer with 47 articles, constituting 5.85% of the total. Among these, 10 journals contributed more than 10 articles each, while the remainder had less than 10 articles. In **Figure 5A**, node size denotes the number of publications, diverse colors indicate different clusters and line width represents relationship strength. In terms of citations, the Journal of Clinical Oncology stands out as the most cited journal, accumulating 2,549 citations with an impressive average citation of 159.3125 in **Table 2**. These top 10 journals span across regions 1, 2, and 3 in the JCI partitions. The Journal of Clinical Oncology holds the highest Impact Factor (IF) at 45.4, with the largest citation volume and average citation volume, underscoring its significant influence in the field.

**Table 2:** Top 10 journals based on publication outputs.

Journal	Publications	Citations	country	IF (2022)	Quartile	H-index
Lymphatic Research and Biology	94	1628	USA	1.400	Q4	58
Supportive Care in Cancer	47	1555	Germany	3.359	Q1	128
Breast Cancer Research and Treatment	44	1753	USA	3.800	Q2	171
Lymphology	44	1293	Germany	2.500	Q3	50
Annals of Surgical Oncology	26	1220	USA	3.700	Q1	192
Journal of Cancer Survivorship	17	598	USA	3.700	Q1	74
Journal of Clinical Oncology	16	2549	USA	45.300	Q1	600
International Journal of Radiation Oncology Biology Physics	14	799	USA	7.000	Q1	268
Cancer	13	596	USA	6.200	Q1	327
Journal of Reconstructive Microsurgery	12	229	USA	3.900	Q2	61

The dual-map overlay analysis depicted in **Figure 5B** illustrates the coverage of all academic journals, mapping the citation paths across various subject areas. Labels on the left side of the dual-map overlay signify the disciplines covered by the citing journals, while labels on the right side represent the disciplines of the cited journals. The majority of journals, originate from the fields of surgery, dermatology, ophthalmology, medicine, medical sciences, and clinical areas, called research frontier. Cited papers predominantly stem from journals in the areas of molecular, biology, genetics, health, nursing, medicine, sports, rehabilitation, referred to as the Knowledge Base. The boundaries between citing and cited journals denote the communication and connection between the two, with node labels indicating the disciplines encapsulated by different journals. The unsloped axis of the ellipse signifies the number of authors involved, while the vertical axis denotes the number of published journals. This dual-map overlay analysis provides insights into the interdisciplinary nature of BCRL-related research and the diverse range of disciplines contributing to and influenced by this field.

### Analysis of authors

**Figure 6:**
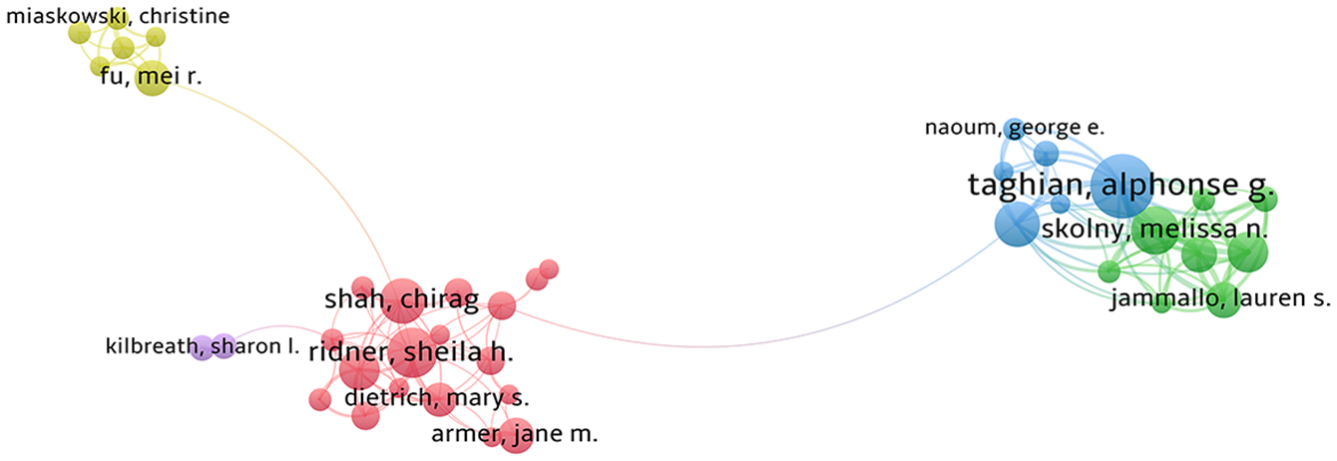
Collaborative network of authors.

**Figure 6** encompasses authors with a minimum of 5 publications, resulting in a total of 40 authors included in the statistical mapping, delineated into 5 clusters with consistent colors indicating the same clusters in **Table 3**. Among these authors, Taghian, Alphonse G., affiliated with Harvard Medical School and Oncology Radiology at Massachusetts General Hospital, emerges as the most prolific collaborators and prolific authors. His research has been instrumental in assessing the risk of lymphedema in breast cancer and standardizing lymphedema assessment, with significant implications for the prevention of BCRL (21–24). One of Taghian’s articles, titled “The Impact of Radiation Therapy on the Risk of Lymphedema After Treatment for Breast Cancer: a Prospective Cohort Study,” garnered the highest citation rate, accumulating 168 citations. The primary focus of this article was on the prospective screening for lymphedema in a large group of breast cancer patients, revealing that regional lymph node radiotherapy (RLNR) significantly increased the risk of lymphedema compared to breast/chest wall radiotherapy alone. The conclusion emphasized the need for clinicians to carefully weigh the potential benefits of RLNR for disease control against the increased risk of lymphedema (25).

**Table 3:** Top 10 authors publishing in the BCRL.

Author	Country	Institution	Publications	Citations	Total link strength
Taghian, Alphonse G.	USA	Harvard Medical School Massachusetts General Hospital	24	1240	105
Ridner, Sheila H.	USA	Vanderbilt Univ, Sch Nursing	17	807	34
Skolny, Melissa N.	USA	Harvard Medical School Massachusetts General Hospital	16	1014	84
Brunelle, Cheryl L.	USA	Harvard Medical School Massachusetts General Hospital	15	575	58
Shah, Chirag	USA	Universitat Siegen Cleveland Clinic Foundation	15	533	25
Boyages, John	Australian	Macquarie University Australian National University	13	307	35
Miller, Cynthia L.	USA	Harvard Medical School Massachusetts General Hospital	13	892	72
Armer, Jane M.	USA	University of Missouri Columbia Ellis Fischer Cancer Center	11	620	8
Fu, Mei R.	USA	George Washington University Rutgers State University System	11	206	13
Jammallo, Lauren S.	USA	Harvard University Massachusetts General Hospital	11	667	66

Ridner, Sheila H., stands out as the author with the second-highest number of publications. Her research, focusing on the assessment of postoperative lymphedema in breast cancer and encompassing literature on health education and nursing care, has achieved notable recognition, reflected in a high citation rate. This suggests that her work has played a pivotal role among numerous authors and has left a substantial impact in the field (26–28). One of Ridner’s seminal articles, titled “Incidence, Treatment Costs, and Complications of Lymphedema After Breast Cancer Among Women of Working Age: A 2-Year Follow-Up Study,” holds the distinction of receiving the highest number of citations, totaling 339. This study delves into the economic burden, incidence of lymphedema, and associated risk factors for BCRL among women of working age (29). The article, published in the Journal of Clinical Oncology, achieved a noteworthy Impact Factor (IF) of 45.4 in 2022, further emphasizing its significance and influence in the field.

### Analysis of keywords and strongest burst keywords

Through the analysis of keyword evolution and frequency changes, it is possible to pinpoint research frontiers and emerging themes. Keywords exhibiting high frequency and centrality values signify research hotspots in the past 20 years, while those with high citation explosiveness can foreshadow future research frontiers.

**Figure 7:**
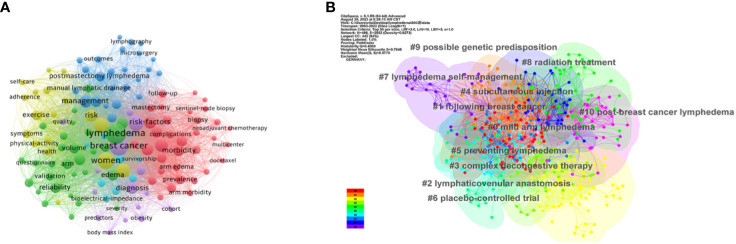
**(A)** Co-occurrence analysis of keywords by overlay visualization. **(B)** Cluster analysis of keywords.

In **Figure 7A**, VOSviewer categorizes 129 keywords into six clusters. As of the end of 2022, “lymphedema,” “breast cancer,” and “women” stand out as the top three high-frequency keywords. **Figure 7B**, utilizing CiteSpace with the classic log-likelihood ratio (LLR) algorithm, results in 11 clusters. With Q = 0.4569 and S = 0.7848, values exceeding Q > 0.3 and S > 0.5 signify significant clustering within the network, indicating consistent literature within each clustered topic. The largest cluster, #0, is centered around “mild arm lymphedema”, with additional subtopics including “lymphaticovenular anastomosis” and “complex decongestive therapy”.

**Figure 8:**
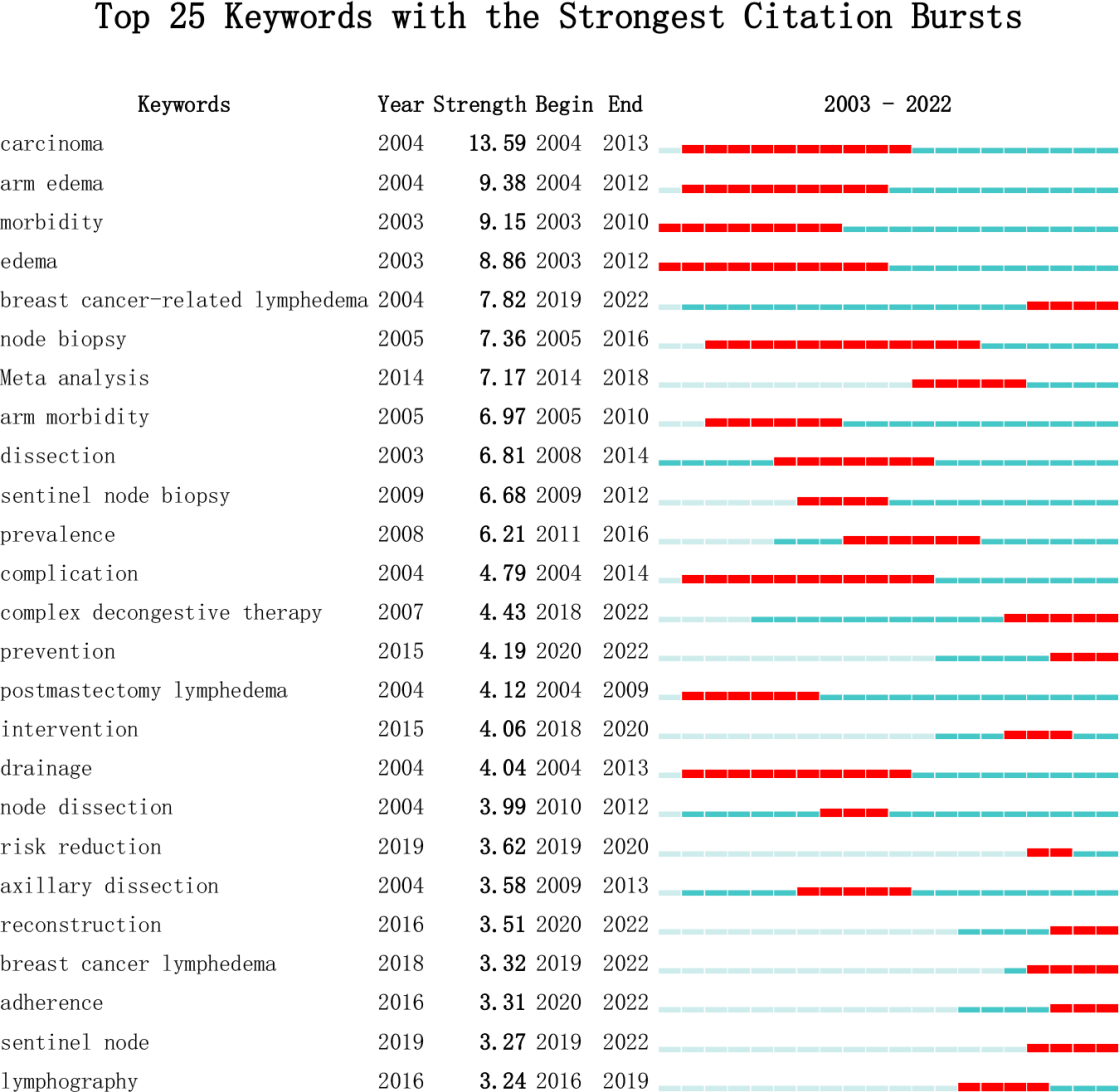
Top 25 Keywords with the Strongest Citation Bursts.

**Figure 8** highlights the top 25 keywords with the strongest citation bursts. The left endpoint of the red line denotes the time of emergence, while the right endpoint indicates the endpoint. Recently emergent keywords underscore current research hotspots, emphasizing “complex decongestive therapy,” “prevention”, and “reconstruction” as key focuses and frontiers in future BCRL-related research.

### Analysis of references

**Figure 9:**
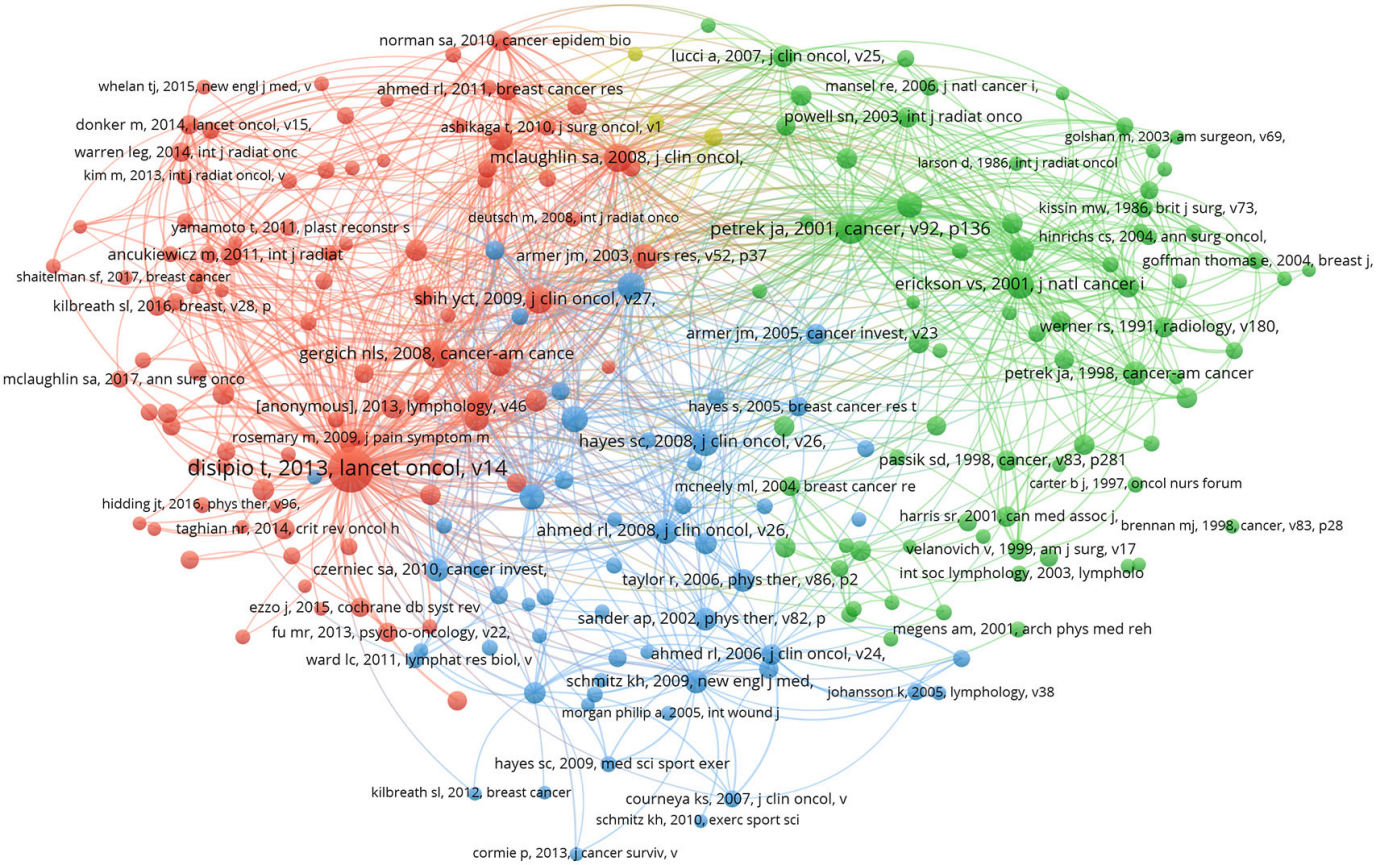
A network diagram of co-cited references.

The reference co-citation map serves as a valuable tool for exploring closely related research topics within the academic field. **Figure 9** includes a total of 10,648 cited articles, and 224 articles meet the criterion of having a minimum number of citations of at least 20. The citation frequency of an article serves as a gauge of its academic significance and impact, with higher citation frequencies indicating increased attention and influence.

**Figure 10:**
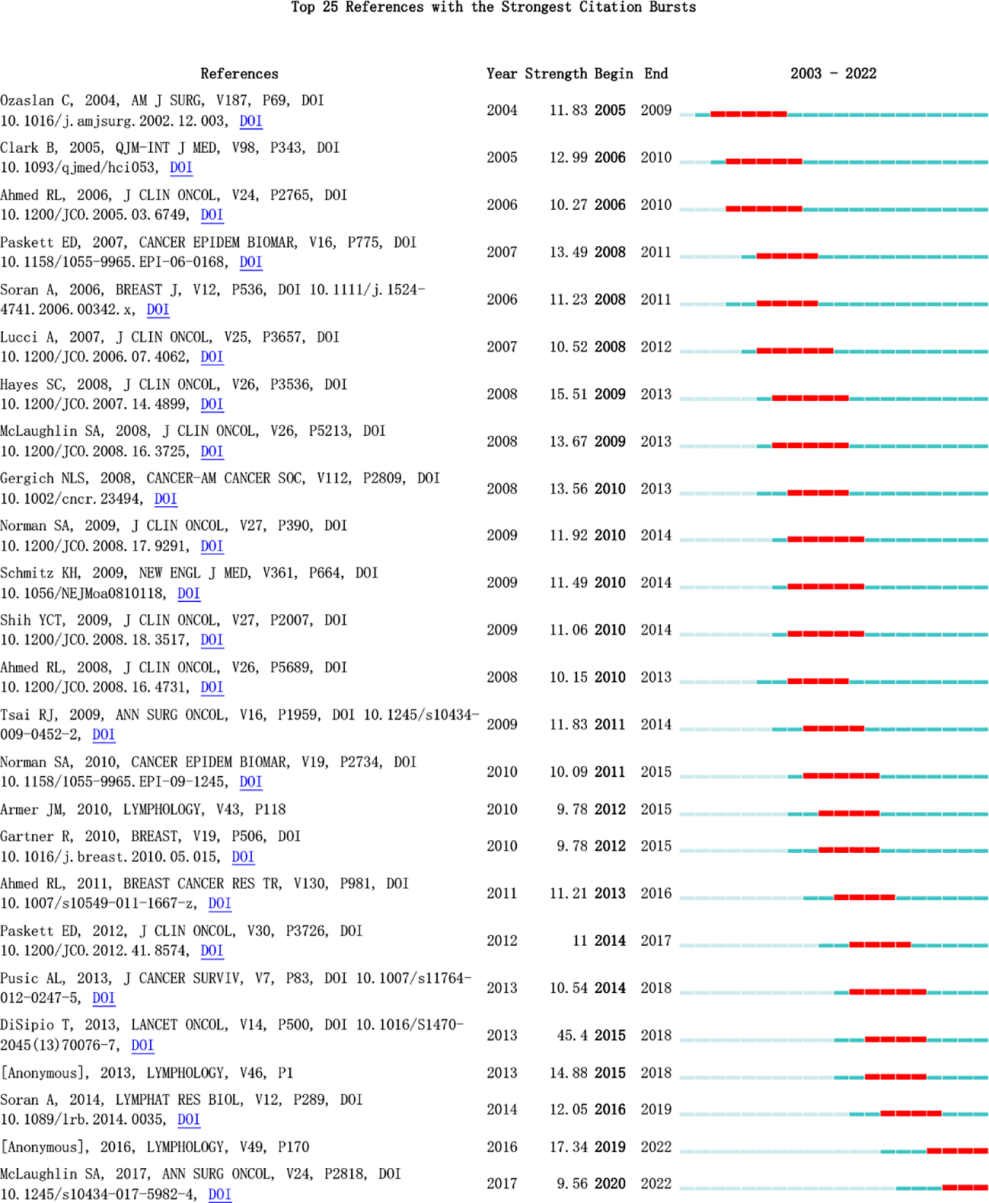
Top 25 References with the Strongest Citation Bursts.

**Figure 10** highlights the top 25 literature with the strongest citation bursts. These bursts, characterized by sudden increases in citation frequency at different times, signify current research hotspots. Analyzing these references provides insights into ongoing research trends and allows for predictions regarding future developments in the field.

In **Table 4**, the most highly cited recent article, published in Lancet Oncology, is titled “Incidence of unilateral arm lymphoedema after breast cancer: a systematic review and meta-analysis.” The authors conducted an analysis that included 72 studies, concluding that the incidence of postoperative lymphedema after breast cancer is approximately 16.6%. However, when considering only 30 prospective studies, the incidence rises to 21.4%, suggesting that prospective studies offer a more accurate reflection of BCRL incidence. The article highlights the impact of the timing of patient inclusion on incidence results, as BCRL manifests acutely for some patients and chronically for others. Factors such as the method of diagnosing lymphedema and the demographic composition of the study population, particularly noting that 13 of the 30 prospective studies focused on North American women, contribute to variations in reported incidence rates. The article underscores the likelihood of underdiagnosis of lymphedema, emphasizing the importance of early detection and management through increased research efforts. Patients can be monitored for lymphedema at 3- to 6-month intervals within the first 2 years of onset, utilizing tools such as bioelectrical impedance spectroscopy (BIS) to measure changes in intra- and extracellular water. The article identifies more extensive surgery (involving the chest wall and axilla) and factors such as overweight or obesity as associated with an increased risk of lymphedema. Modest evidence also supports adjuvant therapies (such as radiation and chemotherapy) and sedentary lifestyles as additional risk factors. The article suggests that future studies could delve deeper into the risk factors for lymphedema development and refine the impact of different treatments on incidence rates (30).

**Table 4:** Top 10 Cited References Related BCRL.

Title	First Author	Citations	Journal	IF(2022)	Publication Year
Incidence of unilateral arm lymphoedema after breast cancer: a systematic review and meta-analysis (30)	DiSipio, T.	1076	Lancet Oncol	51.1	2013
Lymphedema in a cohort of breast carcinoma survivors 20 years after diagnosis (31)	Petrek, Jeanne A.	480	Cancer	6.2	2001
Prevalence of lymphedema in women with breast cancer 5 years after sentinel lymph node biopsy or axillary dissection: objective measurements (32)	McLaughlin, Sarah A.	444	J Clin Oncol	45.3	2008
Arm edema in breast cancer patients (33)	Erickson, VS	418	J Natl Cancer Inst	10.3	2001
Preoperative assessment enables the early diagnosis and successful treatment of lymphedema (34)	Gergich, Nicole L. Stout	349	Cancer	6.2	2008
Incidence, treatment costs, and complications of lymphedema after breast cancer among women of working age: a 2-year follow-up study (29)	Shih, Ya-Chen Tina	340	J Clin Oncol	45.3	2009
Lymphedema after breast cancer: incidence, risk factors, and effect on upper body function (35)	Hayes, Sandra C.	301	J Clin Oncol	45.3	2008
A comparison of four diagnostic criteria for lymphedema in a post-breast cancer population (36)	Armer, Jane M.	279	Lymphat Res Biol	1.4	2005
Lymphedema and quality of life in breast cancer survivors: the Iowa Women’s Health Study (37)	Ahmed, Rehana L.	274	J Clin Oncol	45.3	2008
Lymphedema in breast cancer survivors: incidence, degree, time course, treatment, and symptoms (38)	Norman, Sandra A.	271	J Clin Oncol	45.3	2009

## Discussion

Bibliometrics serves as a systematic tool for delving into academic literature and research findings within related fields. Through literature analysis, it became apparent that despite the substantial number of clinical trials conducted in the realm of BCRL, there exists a dearth of bibliometric studies addressing this specific topic. The application of bibliometric analysis has facilitated an evaluation of the quality of existing studies within the available literature, offering insights into current research activities and shedding light on the level of evidence, impact levels, and potential impact factors. The bibliometric analysis of BCRL has proven invaluable in uncovering trends within the discipline and charting the evolution of research. This, in turn, aids researchers in gaining a more comprehensive understanding of the historical development, current status, and potential future directions of the discipline. Importantly, this wealth of information provides strategic insights for scientists and discipline planners, offering guidance for informed decision-making and shaping the trajectory of future research endeavors.

This study represents the inaugural global bibliometric analysis of BCRL. Utilizing VOSviewer and CiteSpace, we scrutinized 803 articles within the WoSCC database, aiming to delineate research hotspots and forecast future trends spanning the past two decades. The trajectory of publications has exhibited a consistent upward trend since 2003, reaching its zenith in 2018. Extrapolating from current publication patterns, our analysis anticipates a continued steady increase in the number of publications in the foreseeable future.

In the realm of BCRL, the United States emerges as the leading contributor in terms of publications, boasting 286 articles, followed by China with 86. The Mayo Clinic stands out as the most prolific research organization, contributing 32 articles, and notably, many of the institutions with substantial publication numbers are based in the U.S. Among individual authors, Taghian, Alphonse G., holds the top position with the highest number of published articles, totaling 24. His contributions also lead in terms of citations, amassing a remarkable 1,240, indicative of significant impact and fostering collaborative relationships with other scholars. Examining the current classification of BCRL topics reveals that predominant research areas include oncology, physiology, surgery, rehabilitation, and immunology. Based on the insights gleaned from econometric analysis, the study concludes that the primary research hotspots in BCRL presently revolve around complex decongestive therapy, prevention, and reconstruction.

### Complex decongestive therapy

Complex Decongestive Therapy (CDT) represents a comprehensive treatment approach for BCRL and currently stands as the standard in BCRL management. CDT comprises four integral steps, encompassing skin care, freehand lymphatic drainage, compression bandaging, and functional exercise (39–41). A multitude of scholarly investigations has been dedicated to the study of CDT, evaluating its efficacy through various means.Some studies have focused on assessing the effectiveness of CDT using ultrasound (42, 43) or BIS (44) to gauge its impact on edema reduction. Additionally, comprehensive evaluations of CDT’s effects on pain, quality of life, mood, and fatigue in BCRL patients have been conducted (45, 46). Notably, one study highlighted the positive impact of upper body resistance exercise integrated into intensive CDT lymphedema treatment. This intervention was found to enhance arm function and muscle strength without causing an increase in arm volume in patients with BCRL (47).

### Prevention

Surgical prophylaxis in the context of breast cancer involves the identification of sentinel lymph nodes using near-infrared (NIR) fluorescence of indocyanine green (ICG), proving to be a valuable technique. NIR fluorescence holds promise as a key tool for both the prevention and management of lymphedema following axillary dissection for breast cancer. A study by Abbaci et al. (48) encompassed a total of 2016 patients. The application of ICG imaging for axillary reverse labeling was deemed safe for all 951 patients, with arm lymph nodes successfully identified in 80%-88% of those undergoing axillary lymph node dissection. This technique not only serves as a diagnostic tool with high sensitivity and specificity for lymphedema but also finds utility in staging, intraoperative mapping, and patency control of lymphatic foramen anastomosis. The findings underscore the potential of NIR fluorescence with ICG as a multifaceted approach in breast cancer surgery, offering both diagnostic and procedural advantages.

Early screening and prevention with BIS have been the focus of numerous studies aiming to identify individuals at high risk for BCRL. Implementing BIS for early identification and subsequent conservative interventions in high-risk breast cancer patients has shown promising results, notably contributing to a significant reduction in the incidence of BCRL. These findings advocate for the importance of early prospective screening and intervention as effective measures in managing BCRL. The evidence suggests that early detection facilitated by patient-oriented interventions holds the potential to improve patient prognosis and mitigate the risk of persistent BCRL (49–51). This underscores the significance of incorporating BIS into proactive strategies for early screening and intervention, ultimately enhancing patient outcomes in those at heightened risk for BCRL.

Ridner, Sheila H. (28) devised a trial-specific methodology to investigate the impact of early intervention utilizing BIS and to compare it with the effect of combined early intervention with tape measurements. The intervention involved using a compression garment for 4 hours per day over a 12-week period, with the primary endpoint being the incidence of clinical lymphedema. Clinical lymphedema was defined as an incidence requiring CDT, characterized by a ≥ 10% change in volume from the pre-surgical baseline on the tape measure in the high-risk arm. A total of 508 patients participated in this analysis, with 109 (21.9%) triggering prethreshold intervention. In comparison to tape measurements (TM), BIS exhibited a lower trigger rate (15.8% vs. 28.5%, p < 0.001) and a longer trigger time (9.5 months vs. 2.8 months, p = 0.002). Interim findings suggest that post-treatment monitoring with BIS leads to an approximately 10% reduction in the absolute rate of progression of BCRL requiring CDT, representing a clinically meaningful improvement. These results lend support to the concept of utilizing BIS for post-treatment monitoring to detect subclinical BCRL and initiate early intervention.

Exercise and early rehabilitation play pivotal roles in the prevention of BCRL (52–55). Rehabilitative exercises, emphasizing mobility, and physical activities focusing on strength are actively encouraged to mitigate the risk of BCRL. Incorporating these measures into the management and care of individuals with breast cancer not only supports overall physical well-being but also serves as an integral aspect of preventive strategies against lymphedema-related complications.

Weight control is recognized as a crucial aspect in preventing BCRL (56–58). Maintaining a stable weight and avoiding overweight and obesity are advised preventive measures. For individuals who are overweight or obese, offering dietary guidance to reduce body mass index is recommended. Implementing a structured exercise program and adhering to a prescribed diet are significant contributors to achieving successful weight loss and its maintenance. Obese breast cancer survivors may particularly benefit from weight loss interventions, not only in reducing their risk of lymphedema but also in enhancing their overall health (59). Emphasizing weight control strategies is integral to comprehensive care for breast cancer patients, contributing to both lymphedema prevention and broader health outcomes.

### Reconstruction

In a clinical study conducted by CARD A et al. (60) involving 574 cross-matched patients, 78 individuals (6.8%) developed lymphedema, with 21 cases occurring in reconstructed breasts and 57 in non-reconstructed breasts. Notably, patients who did not undergo reconstruction were significantly more likely to develop BCRL (9.9% vs. 3.7%, p < 0.001). Furthermore, the onset of lymphedema occurred significantly later in reconstructed patients compared to non-reconstructed patients (p < 0.001). This study suggests that patients who underwent breast reconstruction exhibited a lower incidence and delayed onset of breast cancer-associated lymphedema compared to those who underwent mastectomy alone.

In surgical interventions, lymph node or lymphatic vessel reconstruction is often incorporated alongside breast reconstruction. Lymphatic venous anastomosis (LVA) stands out as a minimally invasive procedure designed to redirect lymph to the dermal venous drainage system. This technique demonstrates notable improvements in volume and, in specific cases, may obviate the need for compression therapy. LVA not only significantly enhances quality of life but also positively impacts the patient’s mood and self-perception (61). Vascularized lymph node transfer (VLNT) represents another microsurgical approach frequently combined with autologous free flap breast reconstruction. This technique aims to address lymphedema and brachial plexus neuropathy while minimizing the risk of cellulitis (62). The synergistic application of LVA and VLNT, possibly in conjunction with other methods, optimizes their effectiveness. Moreover, vascularized lymphatic vessel transfer (VLNT) involves harvesting specific lymphatic vessels while preserving lymph nodes at the donor site. VLNT is typically reserved for patients lacking functional lymph nodes in the affected limb and for whom lymphatic vessels are no longer amenable to LVA treatment. Current literature supports the efficacy of VLNT, indicating a significant 40% reduction in BCRL volume in approximately 90% of patients (63, 64).

Two relatively recent surgical strategies include immediate lymphatic reconstruction (ILR) during axillary lymph node dissection and the combination of vascularized lymph node transfer with Deep Inferior Epigastric Artery (DIEP) flap breast reconstruction. Immediate Lymphatic Reconstruction (ILR), also known as the Lymphatic Microsurgical Prophylactic Healing Approach (LyMPHA), involves performing prophylactic lymphovenous anastomosis at the time of axillary lymph node dissection (ALND). While several techniques have been proposed for managing lymphedema after its onset, the prophylactic application of ILR aims to decrease the risk of development to 6.6% (65). Furthermore, the combination of vascularized lymph node transfer with DIEP flap breast reconstruction offers notable improvements in lymphedema-related quality of life, even without a reduction in volume difference. This approach also results in reduced dependence on compression garments and a decreased requirement for physical therapy (66).

### Limitation

Several limitations of this study should be acknowledged. Firstly, we exclusively relied on data from the Web of Science (WOS) and did not consider literature from other databases. Secondly, due to time constraints, literature outside the specified period was excluded. Additionally, software limitations may have prevented the modification of case formats and abbreviations, and the settings of thresholds and cropping methods might have led to the inadvertent exclusion of some data.

### Conclusion

Our comprehensive bibliometric analysis of Breast Cancer-Related Lymphedema (BCRL) research from 2003 to 2022 offers valuable insights into the evolving landscape of this field. The study revealed a consistent upward trend in BCRL publications, peaking in 2018, with the United States emerging as the predominant contributor. Collaborative networks among researchers and institutions worldwide underscore the global nature of BCRL research. Noteworthy research hotspots identified encompass preventive strategies, complex decongestive therapy, and reconstructive interventions. These findings underscore the multidimensional approach required to address the complexities of BCRL management effectively.

In summary, this study provides a comprehensive overview of BCRL research trends and collaborations globally. It serves as a foundational resource for researchers, clinicians, and policymakers, fostering evidence-based practices and interventions for BCRL in the future. By emphasizing an evidence-based approach, this study aims to provide valuable insights into the field and offer an exploratory analysis to further advance BCRL research.

## Data availability statement

The original contributions presented in the study are included in the article/supplementary material. Further inquiries can be directed to the corresponding authors.

## Author contributions

HJ: Writing – original draft, Writing – review & editing, Conceptualization, Data curation, Formal analysis, Funding acquisition, Investigation, Methodology, Project administration, Resources, Software, Supervision, Validation, Visualization. FF: Writing – review & editing, Conceptualization, Data curation, Formal analysis, Funding acquisition, Investigation, Methodology, Project administration, Resources, Software, Supervision, Validation, Visualization, Writing – original draft. FH: Conceptualization, Formal analysis, Investigation, Software, Writing – original draft, Writing – review & editing. JL: Conceptualization, Investigation, Software, Supervision, Writing – original draft, Writing – review & editing. YL: Conceptualization, Data curation, Investigation, Methodology, Software, Writing – original draft, Writing – review & editing. CL: Conceptualization, Data curation, Formal analysis, Funding acquisition, Investigation, Methodology, Software, Supervision, Writing – original draft, Writing – review & editing. LH: Data curation, Software, Writing – original draft, Writing – review & editing. BL: Conceptualization, Funding acquisition, Investigation, Visualization, Writing – original draft, Writing – review & editing.
